# Uptake of Rabies Control Measures by Dog Owners in Flores Island, Indonesia

**DOI:** 10.1371/journal.pntd.0003589

**Published:** 2015-03-17

**Authors:** Ewaldus Wera, Monique C. M. Mourits, Henk Hogeveen

**Affiliations:** 1 Animal Health Study Program, Kupang State Agricultural Polytechnic, West Timor, Indonesia; 2 Business Economics Group, Wageningen University, Wageningen, The Netherlands; The Global Alliance for Rabies Control, UNITED STATES

## Abstract

**Background:**

Rabies has been a serious public health threat in Flores Island, Indonesia since it was introduced in 1997. To control the disease, annual dog vaccination campaigns have been implemented to vaccinate all dogs free of charge. Nevertheless, the uptake rate of the vaccination campaigns has been low. The objective of this paper is to identify risk factors associated with the uptake of rabies control measures by individual dog owners in Flores Island.

**Methodology/principal findings:**

A total of 450 dog owners from 44 randomly selected villages in the Sikka and Manggarai regencies were interviewed regarding their socio-demographic factors, knowledge of rabies, and their uptake of rabies control measures. The majority of dog owners surveyed (>90%) knew that rabies is a fatal disease and that it can be prevented. Moreover, 68% of the dog owners had a high level of knowledge about available rabies control measures. Fifty-two percent of the dog owners had had at least one of their dogs vaccinated during the 2012 vaccination campaign. Vaccination uptake was significantly higher for dog owners who resided in Sikka, kept female dogs for breeding, had an income of more than one million Rupiah, and had easy access to their village. The most important reasons not to join the vaccination campaign were lack of information about the vaccination campaign schedule (40%) and difficulty to catch the dog during the vaccination campaign (37%).

**Conclusions/significance:**

Dog owners in Flores Island had a high level of knowledge of rabies and its control, but this was not associated with uptake of the 2012 vaccination campaign. Geographical accessibility was one of the important factors influencing the vaccination uptake among dog owners. Targeted distribution of information on vaccination schedules and methods to catch and restrain dogs in those villages with poor accessibility may increase vaccination uptake in the future.

## Author Summary

Rabies has been a serious public health threat in Flores Island since its introduction in 1997, with significant economic consequences for the government due to the cost of providing vaccines for humans and dogs. Vaccination of dogs against rabies offers a safe and effective means to prevent rabies in humans in the long term. The local government of Flores Island has implemented dog vaccination campaigns throughout the island, free of charge for all dog owners. However, the uptake rate of vaccinating dogs is too low to control the disease. Identifying factors associated with the dog owners’ decisions to vaccinate their dogs provides information to policy makers to increase vaccination uptake in the future. This study evaluated the impact of rabies knowledge and socio-demographic factors of dog owners on the uptake of the 2012 dog vaccination campaign. Overall, the uptake rate of the 2012 vaccination campaign was low. Levels of knowledge about rabies and its control were high, but not associated with uptake of vaccination. Geographical accessibility was significantly associated with vaccination uptake. Targeted distribution of information in those villages with poor accessibility may increase vaccination uptake in the future. Information should cover vaccination schedules and methods to restrain dogs.

## Introduction

Rabies still poses a significant health problem in many countries of the world, despite it being a vaccine-preventable disease in dogs and humans [1]. Approximately 55,000 people around the world die each year due to rabies, with 45% of these cases occurring in the South East Asian region [2]. Within this region, Indonesia has the fourth largest number of human rabies cases after India, Bangladesh and Myanmar, with 150–300 cases reported per year [2].

The first occurrence of rabies in Indonesia was reported in 1889 [3]. Since its introduction, rabies has posed a serious public health threat with significant economic consequences to society [4]. The national strategic plan of Indonesia emphasizes the control of rabies as a policy priority, aiming for eradication by the year 2020 [5].

Flores Island is located in the eastern part of Indonesia and covers an area of 15,624 km^2^ [6]. The island is divided into eight regencies, with a human population of more than 1.8 million [7] and a dog population greater than 0.2 million [4]. Many of the rural areas on the island are only accessible by foot or with high-clearance vehicles, motor bikes, or horses [8]. The main socio-economic activity on the island is agriculture (production of coconut, corn, groundnut, cocoa, coffee, potato, and paddy), in which dogs are used to guard the crops [9]. Most dogs are owned and roam freely day and night. Although Indonesia is predominantly Muslim (practicing the Islamic principles in which it is prohibited to eat dog meat or to keep dogs inside the house), the majority of people in Flores are Catholic. Dogs have a high cultural and economic value in Flores Island, as they provide a source of animal protein in addition to their guarding capacities. Dog meat is a popular menu item in certain traditional ceremonies of the island [9].

On Flores Island, the first cases of dog rabies were officially confirmed in April 1998 in the regency of East Flores [10]. The introduction of the disease was traced back to three suspected rabid dogs that were brought from Buton Island by a fisherman in September 1997 [10]. Despite the initial control measures applied, which entailed the culling of the entire dog population in and around the affected villages (1998–1999), rabies spread to other regencies of the island [11]. In response, the Flores Island government have implemented a comprehensive control campaign since 2000. This control campaign is based on a combination of control measures, including mass vaccination of dogs, culling of roaming dogs, placing imported dogs in quarantine, and giving pre- and post-exposure treatment to humans. Complementary control measures include investigation of dog bites, diagnostic testing of suspected rabid dogs, and tracing of human contacts with rabid dogs. However, this campaign has not yet been successful in eliminating rabies from Flores Island.

Thousands of people bitten by dogs are looking for post-exposure treatment each year, resulting in a large economic cost for both government and local communities. The annual cost of rabies control efforts in Flores Island has been estimated to exceed US$ 1.0 million [4]. The impact on public health is difficult to measure. Until 2012, 96 human cases of rabies were officially registered by the Public Health Department, with the highest number of cases in Manggarai regency (27 cases), followed by Sikka (22 cases), Ngada (16 cases), West Manggarai (11 cases), East Manggarai (8 cases), East Flores (6 cases), Nagakeo (4 cases), and Ende (2 cases) [12]. However, these numbers do not reflect the real human rabies burden in Flores, as the data only capture the number of rabies patients who visited hospitals or public health centers during the period that rabies was clinically manifest. The number of human cases reported by the Husbandry Department of East Nusa Tenggara Province was more than two times higher (228 cases) [13] than the 96 cases officially recorded by the Public Health Department [12].

During the last thirteen years, the regencies on Flores Island have implemented annual dog vaccination campaigns using Rabivet Supra 92 [4]. Although vaccination is compulsory for all dogs (Manggarai Regency Law, number 6, year 2003), it is difficult to enforce due to the absence of a proper registration system and the lack of resources to catch and restrain dogs. Vaccination is therefore only feasible with the support of the dog owner, who presents and restrains the dogs for vaccination. To increase vaccination coverage, regencies have offered dog owners the vaccination of their dogs for free. Moreover, the vaccine has been delivered using a ‘house-to-house’ approach, undertaken by the local authority [4] to directly persuade dog owners to vaccinate their dogs. The ‘house to house’ vaccination approach is a method in which the vaccination teams visit the dog owners at their own homes. As the vaccination team are not equipped to handle roaming dogs, dog owners need to catch and restrain their dogs themselves. Because the dogs in Flores Island are not used to being restrained, it is expected that ‘house-to-house’ vaccination campaigns result in a higher vaccination coverage than central point vaccination campaigns [14]. Moreover, ‘house-to-house’ campaigns put more social pressure on dog owners to vaccinate than central point campaigns. Nevertheless, the uptake rate of the dog vaccination measure has been low, with an average vaccination coverage of around 53% of the registered dogs during the 2000–2011 vaccination campaigns [4]. This value is lower than the 70% coverage of the complete dog population, which is recommended to maintain the control of rabies between annual vaccination campaigns [1].

Although there are publications describing the uptake of dog vaccination campaigns in developing countries [14,15,16,17,18,19,20], none of these studies have focused on the situation of rabies in Flores Island, nor evaluated the impact on the uptake of vaccination of the socio-demographic characteristics of dog owners and their knowledge of rabies. An understanding of this impact is essential to support policy decisions about rabies control in the future. The objective of this paper is to identify risk factors associated with the uptake of rabies control measures by dog owners in Flores Island, Indonesia. This is achieved by undertaking an extensive survey among dog owners in the regencies of Sikka and Manggarai. Risk factors concern socio-demographic factors and the level of rabies knowledge of dog owners. Special emphasis is given to risk factors associated with the uptake of the vaccination campaign in 2012.

## Materials and Methods

### Study area

An extensive survey was conducted among dog owners in the regencies of Sikka and Manggarai during January and February 2013. The regencies were selected because of the high prevalence of human rabies and the control legislation in place. Sikka relies on the national rabies control campaign, whereas Manggarai has a local control legislation in place [4]. Based on this local legislation, Manggarai has been applying additional control measures (such as culling) alongside the nationally recommended vaccination control campaign. The regencies have similar sized populations, 300,301 inhabitants in Sikka and 292,037 inhabitants in Manggarai (census data of 2010) [7]. There are no officially registered data available on the number of villages and the number of households owning dogs, nor on the size of the dog population. During the ‘house-to-house’ rabies vaccination campaign in 2012, 351 villages in Sikka and 162 villages in Manggarai were involved. All households in these villages were visited by the local authorities to vaccinate the dogs for free. Given the number of dogs registered during this vaccination campaign, the number of dogs is estimated at 37,000 in Sikka and 6,675 in Manggarai. The difference in the number of dogs per regency is a result of the culling measures that were implemented in Manggarai.

### Sample size and design

The minimum sample size required to estimate the proportion of dog owners vaccinating their dogs was based on the conservative assumption that 50% of dog owners vaccinated at least one of their dogs (*p*_exp_), with a 5% error in estimate (*d*) and a 95% confidence interval. Given the standard power calculation:

$$n=\frac{1.96^{2}\times p_{\text{exp}}\times \left(1-p_{\text{exp}}\right)}{d^{2}}$$ (1)

The required sample was a minimum of 385 dog owners. The sample size was increased to 450 dog owners to account for incomplete interviews, with 300 dog owners sampled in Sikka and 150 in Manggarai. The relative size of the samples in Sikka and Manggarai reflected the difference in the number of villages involved in the 2012 vaccination campaign, which was 351 villages in Sikka and 162 villages in Manggarai.

Dog owners were selected from the villages included in the 2012 vaccination campaign. A random order of villages for the survey was obtained for each regency by randomly ranking the villages involved in the 2012 vaccination campaign. Subsequently, villages were visited in the order of this ranking until the predefined sample sizes (300 dog owners in Sikka and 150 dog owners in Manggarai) were reached. A total of 44 villages were visited, with 27 villages in Sikka and 17 villages in Manggarai. The following process was carried out in each of the 44 villages in the survey. Firstly, the village leader was approached to inform him about the study and to seek permission to carry out the study in his village. Subsequently, 7 to 15 dog owners (respondents) aged 18 years or older were selected per village. The number of dog owners to be interviewed was predefined for each village and proportional to the number of dog owners involved in the rabies vaccination campaign of 2012. Due to the lack of registration data on households, the first respondent in each village was chosen by chance by spinning a pen at the center of the village [20]. The direction of the pen tip determined the first household/respondent to be interviewed. In case there were no dogs or adult persons present in the house, the next household was selected [21]. Subsequent respondents were selected from the closest neighboring households that owned dogs.

The questionnaire interviews were conducted by two survey teams (one team per regency) assisted by local people with knowledge of the local languages (Sikka, Lio, and Manggarai) as well as Bahasa Indonesia. Prior to each individual interview, the purpose of the survey was explained to the respondents. A verbal informed consent (permission to carry out the interview) was obtained from the dog owners before the interview was conducted. The interviews generally took place between 8 a.m. and 6 p.m., from Mondays to Saturdays. When there were not enough participants available in a village due to the absence of dog owners, the interviews were subsequently administrated in the early morning or evening of the next day. Daily evening briefings among the survey team members ensured interview consistency. Obtained data were entered in Data Editor of SPSS software version 19.

### Questionnaire design

The questionnaire was designed after an extensive literature review of previous survey studies, which focused on either the level of rabies knowledge or the uptake of rabies control measures in dogs and humans [18,20,22,23].

The questionaire contained open and closed questions, which were divided into four sections: (1) socio-demographics of dog owners, (2) knowledge of rabies and its control measures (3) uptake of rabies control measures, and (4) reasons for joining or not joining the rabies vaccination campaign of 2012.

**1) Socio-demographics.** Questions on the socio-demographics of dog owners covered personal characteristics, characteristics of the household, characteristics of the dogs and reasons for keeping them, and one characteristic of the village. Personal characteristics included gender, age, education level, occupation, income, and religion. Characteristics of the household included the size of the household and presence of children in the household. Characteristics of the dogs included whether female for breeding or male dogs were kept, reasons for keeping dogs, and the economic value of dogs. The characteristic of the village concerned the accessibility of the village. This was assessed for each village according to the type of road infrastructure between the village and the main road. Three categories were used to stratify accessibility. Villages located along the provincial road connecting West to East Flores Island were categorized as villages with ‘good accessibility’, as these villages have easy access to frequent public transportation. Villages located more than 3 km from the main road, which have less frequent access to public transportation, were categorized as villages with ‘average accessibility’. Villages that can only be reached by foot or motorcycle, which have no public transportation facility, were categorized as villages with ‘poor accessibility’.

**2) Knowledge of the risk, prevention in humans, and control of rabies.** The knowledge of the dog owners about the risk of rabies to humans was assessed using the following two ‘yes’ or ‘no’ questions, as modified from Tenzin et al. [22]: (1) “*do you know that rabies is a fatal disease in humans*?*”* and (2) “*do you know that rabies in humans can be prevented*?*”*. To assess the level of knowledge of rabies control measures, a subsequent question was posed to those dog owners who responded positively to the second question: “*which measures are known to you in order to prevent rabies in humans*?*”*. The intention of this question was to evaluate the level of knowledge about the *range* of control measures that could prevent rabies in humans. This knowledge does not necessarily reflect an understanding of the efficacy of the control measures. The respondents’ answers to the question were classified as either correct or incorrect based on scientific evidence [24,25]. Answers were considered scientifically correct if the control measures mentioned by the respondents were in line with those recommended by the WHO [1,26] and OIE [27]. These recommendations consist of: (1) vaccination injections before exposure (pre-exposure treatment), (2) cleaning wound after being bitten, (3) injection of human rabies vaccines and/or immunoglobulin after exposure (post-exposure treatment), (4) vaccination of dogs, (5) dog movement restrictions, and (6) leashing of dogs. Each corresponding answer was given a score of 1. Answers that were not based on scientific evidence (e.g., prayer and traditional medicine) were given a score of 0. In addition, as culling of dogs is a control measure within Manggarai regency law (number 6, year 2003), an additional score of 1 was given to those respondents who reported culling as a control measure. Within Mangarai regency law, this culling refers to the culling of dogs that are aggressive and tend to bite and culling of roaming dogs in newly infected villages and public areas regardless of their health status. The total score per respondent (range of 0–7) was subsequently categorized into a binary variable, by defining one category to indicate total scores lower than the median score of all answers and another category to indicate total scores equal to or higher than the overall median score [22].

**3) Uptake of rabies control measures.** To obtain information about the uptake of rabies control measures, dog owners were asked about the measures they had adopted during the period 1999–2012. With respect to the control measures in dogs, dog owners were specifically questioned about the uptake of vaccination and culling. In addition, the uptake of a complementary rabies control measure was also specified, namely castration of male dogs as part of dog-population management. We did not consider the option to sterilize female dogs, assuming that very few female dogs will be sterilized due to the lack of animal health facilities and the costs involved.

Concerning the control measures in humans, dog owners who indicated that they had experienced a bite incident in their family were asked about their uptake of post-exposure treatments (wound cleaning, rabies immunoglobulin injection, and series of rabies vaccine injections). The uptake items were recorded into dichotomous variables for subsequent data analysis (1 = ‘Uptake’ and 0 = ‘No uptake’).

**4) Reasons for (not) joining the rabies vaccination campaign of 2012.** In the final item, motives for joining or not joining the 2012 dog vaccination campaign were explored with open questions. The question about the motives for joining was posed to the dog owners who had vaccinated their dogs, allowing them to mention multiple reasons. The question about motives for not joining was posed to those who had not vaccinated their dogs and allowed them to indicate only the main reason.

The questionnaire was developed in English and translated to Bahasa Indonesia. It was pre-tested by a focus group, consisting of 4 veterinarians, 4 veterinarian assistants/vaccinators, a husbandry officer of the Animal Health and Husbandry Department of Sikka, and by five pilot interviews [22] with dog owners from a village in Sikka. The final questionnaire was revised based on the pre-test and pilot interviews to improve clarity and interpretation [22]. An interview took approximately 45 minutes to complete the questionnaire.

### Statistical analysis

Descriptive statistics were used to summarize the dog owners’ responses for each of the four survey items. Differences in the proportions of dog owners with knowledge of rabies (yes/no), knowledge of control measures (high/low), and uptake of the 2012 vaccination campaign (yes/no) were tested using the Chi-square test. The associations of the socio-demographic factors with the levels of knowledge (i.e. about the risk of the disease, prevention in humans, and control measures) and the uptake of the 2012 vaccination campaign were assessed using univariable logistic regression analyses. The effect of the knowledge level of dog owners on the uptake of the 2012 dog vaccination campaign was also explored using an univariable logistic regression analysis. Four multivariable logistic regression analyses were conducted to determine the independent contribution of each of these variables to the outcome (e.g. uptake of the 2012 dog vaccination campaign) after adjusting for other variables [28,29]. All independent variables, which had p-values of less than 0.25 in the univariable analyses were subsequently included in the initial models for the multivariable analyses [30].[22,31]. Prior to the multivariable analyses, Spearman’s rank correlation coefficient (ρ) was calculated to check for multicollinearity between the independent variables selected from the univariable analyses. Multicollinearity was considered to be present at ρ>0.7. The final multivariable logistic models were derived by backward stepwise elimination of variables with a p-value greater than 0.05. The Hosmer-Lemeshow goodness-of-fit test was performed to determine the fit of the final models with the data [30].

The multivariable models are represented by the logit formula:

$$\text{ln}\left(\frac{v}{1-v}\right)=\beta_{0}+\beta_{1}x_{1}+\beta_{2}x_{2}+.....+\beta_{p}x_{p},$$ (2)

where $\text{ln}\left(\frac{v}{1-v}\right)$ is the log of the odds of the outcomes (i.e., having knowledge about rabies and the risk it poses for humans or not, having a high or low level of knowledge about rabies control measures, and having participated in the 2012 vaccination campaign or not, represented by (*v*) and (1 − *v*), respectively), *β*_0_ is the estimated intercept, and *β*_(1)_ ,.., *β*_(*p*)_ represent the regression coefficients of each independent variable included in the model. Exponentiation of these regression coefficients (*e* ^*β*_(1)_,...,*β*_(*p*)_^) gives the odds ratios (OR) for each independent variable. SPSS version 19 was used for the analysis of all the data.

## Results

### Socio-demographic characteristics of the dog owners

A total of 463 households were visited. Of these households, 5 dog owners (in Sikka) refused to be interviewed and 8 dog owners (3 in Sikka and 5 in Manggarai) were not at home during the time of the interviews. The socio-demographic characteristics of the 450 respondents are shown in Table 1. The majority of the respondents was male (67%), aged between 18–45 years (56%), and had children in the household (84%). Most respondents were farmers (79%) and of catholic religion (99%). Of the 450 respondents, almost 50% had attended or graduated from elementary school. The median numbers of humans and dogs per household were 5.0 humans (mean 5.3; range: 1–11) and 2.0 dogs (mean 2.2; range: 1–12). The majority of dog owners (68%) indicated that they kept dogs to guard their house and property, and to chase away wild animals that destroy their crops.

**Table 1:** Distribution of socio-demographic characteristics of the surveyed dog owners in Flores Island in relation to their knowledge of rabies and its control and their uptake of the 2012 vaccination campaign.

Variables	*Rabies is a fatal disease in humans* (N = 450)			*Rabies in humans can be prevented* (N = 450)			Knowledge of rabies control measures (N = 403)*			Uptake 2012 vaccination campaign (N = 450)		
	Yes	No		Yes	No		High	Low		Yes	No	
	n	n	p-value	n	n	p-value	n	n	p-value	n	n	p-value
Regency:			**0.000**			**0.000**			**0.141**			**0.000**
Sikka	289	11		284	16		198	86		189	111	
Manggarai	126	24		119	31		74	45		45	105	
Gender:			**0.096**			**0.153**			0.619			**0.030**
Male	275	28		267	36		178	89		147	156	
Female	140	7		136	11		94	42		87	60	
Age:			0.570			0.303			0.925			0.850
18–45 years	234	18		229	23		155	74		132	120	
>45 years	181	17		174	24		117	57		102	96	
Highest education:			**0.160**			**0.001**			0.851			0.720
None	38	7		33	12		21	12		20	25	
Elementary school	206	18		201	23		139	62		117	107	
Junior high school	77	5		76	6		49	27		45	37	
Senior high school/University	94	5		93	6		63	30		52	47	
Occupation:			0.940* *			0.647**			0.967			**0.190**
Farmer	327	29		316	40		214	102		179	177	
Public service	20	1		20	1		13	7		10	11	
Others	68	5		67	6		45	22		45	28	
Monthly income of dog owners (in Rupiah(Rp)^1^)***:			0.006**			**0.129**			**0.024**			**0.020**
< 500,000	227	10		212	25		148	64		137	100	
500,000–1,000,000	131	13		133	11		80	53		62	82	
> 1,000,000	55	10		54	11		43	11		33	32	
Religion^2^:			1.000* *			1.000**			. 000**			0.677**
Islam	2	0		2			2	0		1	1	
Protestant	4	0		4			3	1		1	3	
Catholic	409	35		397	47		267	130		232	212	
Number of people per household:			0.607			0.982			**0.022**			0.848
≤ Two	31	1		28	4		16	12		17	15	
Three	42	3		40	5		23	17		20	25	
Four	68	8		69	7		45	24		42	34	
Five	98	6		94	10		76	18		54	50	
≥Six	176	17		172	21		112	60		101	92	
Having children in the household:			**0.029**			0.307			0.308			0.590
Yes	345	34		337	42		231	106		195	184	
No	70	1		66	5		41	25		39	32	
Having family member previously bitten by dogs:			0.396			**0.040**			0.923			0.684
Yes	84	5		85	4		57	28		48	41	
No	331	30		318	43		215	103		186	175	
Having female dogs for breeding^3^:			0.506			**0.001**			0.341			**0.000**
Yes	214	16		217	13		142	75		145	85	
No	201	19		186	34		130	56		89	131	
Having male dogs:			**0.099**			0.609			0.626			**0.130**
Yes	237	25		233	29		155	78		144	118	
No	178	10		170	18		117	53		90	98	
Primary function of dogs:			0.024**			0.031**			**0.018**			**0.060**
Economy	89	6		87	8		60	27		13	14	
Source of protein	19	3		17	5		12	5		45	50	
Guard of house/property	238	15		233	20		167	66		8	14	
Hunter (chase away) wild animals	43	10		43	10		23	20		146	107	
Traditional ceremony	26	1		23	4		10	13		22	31	
Economic value of dogs**** (in Rupiah (Rp)^1^ per dog):			0.001**			0.000**			0.619			**0.000**
≤ 250,000	89	11		84	16		57	27		45	55	
>250,00–500,000	309	17		303	23		206	97		186	140	
> 500,000	17	7		16	8		9	7		3	21	
Having livestock:			0.334			0.615			**0.077**			**0.010**
Yes	315	24		305	34		213	92		188	151	
No	100	11		98	13		59	39		46	65	
Accessibility of the village:			0.953			0.505			**0.131**			**0.002**
Poor	135	12		130	17		81	49		60	87	
Average	201	16		198	19		134	64		119	98	
Good	79	7		75	11		68	18		55	31	

*The question was only posed to dog owners who knew that “rabies in humans could be prevented” (n = 403).

**Fisher x^2^ square test.

***4 missing values.

****The economic value of dogs was based on the owners’ estimation.

^1^The currency rate when the study was conducted, 1 February 2013: 1US$ = Rp 9.651.

^2^The actual influence of the religion variable could not be quantified due to the small sample size in some categories.

^3^Female dogs that had been giving birth during their life.

p-value shown in bold represents p<0.25.

Differences were tested with a Chi square test.

### Knowledge of the risk, prevention in humans, and control of rabies

The majority of the dog owners surveyed in Sikka and Manggarai regencies agreed with the statement that *“rabies is a fatal disease in humans”* (92%). Agreement with this statement was significantly different (p<0.05) for regency, income, presence of children in the household, primary function of dogs, and economic value of dogs (Table 1).

**Fig 1:**
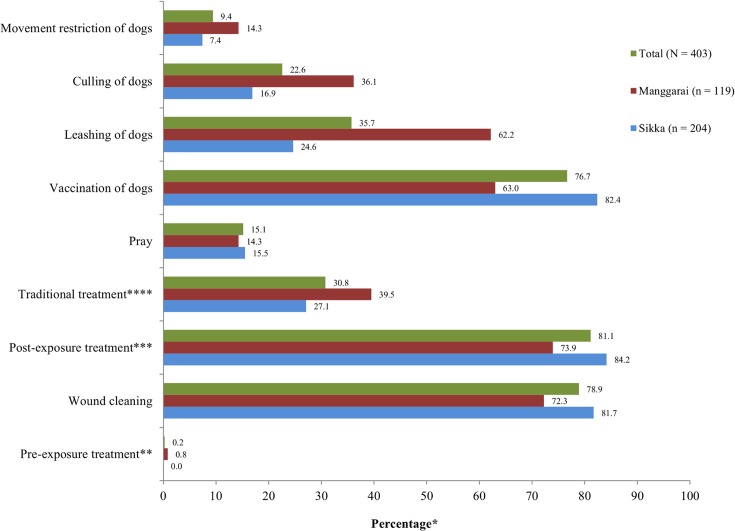
Rabies control measures known by dog owners who agreed with the statement that *“rabies in humans can be prevented”* (N = 403). *Dog owners were allowed to provide more than one response; therefore, percentages of reasons do not sum to 100%; **A series of vaccination injections before exposure; ***A series of vaccination injections and/or immunoglobulin injection after exposure; ****Treatment relying on healers, herbs, etc.

Four hundred and three dog owners (90%) agreed with the statement *“rabies in humans can be prevented”*. Agreement was significantly different (p<0.05) for regency, education level of dog owners, having a family member previously bitten by dogs, having female dogs for breeding, primary function of dogs, and economic value of dogs (Table 1). The preventive measures that were most frequently known by the dog owners, who agreed with the statement that rabies in humans can be prevented (n = 403), were vaccination and/or immunoglobulin injection (81%) and wound cleaning (79%) (Fig. 1). Other indicated control measures included dog vaccination (77%), leashing of dogs (36%), traditional treatment (31%), and prayer (15%). The total number of scientifically correct measures indicated per dog owner varied between 0 and 6, with a median of 3. The majority of the dog owners (68%) mentioned 3 or more measures, indicating a relatively high level of knowledge about rabies control measures (Table 1). Only four respondents (1%) indicated traditional treatment and prayer as the only means to prevent rabies in humans. The level of knowledge of control measures differed significantly (p<0.05) by income, religion, number of people per household, and primary function of dogs. The level of knowledge was not significantly different between regencies (p>0.05).

**Factors related to knowledge of the risk, prevention, and control of rabies.** The factors regency, having male dogs, and economic value of dogs were significantly related with knowledge of the risk of rabies in humans (Table 2). Dog owners living in Sikka were more aware about the risk of rabies in humans (OR = 5.55; 95% CI = 2.33–13.18) compared to dog owners living in Manggarai. Dog owners having male dogs had lower odds of having knowledge of the risk of rabies in humans (OR = 0.41; 95% CI = 0.18–0.96) compared to their counterparts. Dog owners who kept dogs with an average economic value between Rp250,000 and Rp500,000 per dog were more likely (OR = 2.74; 95% CI = 1.14–6.59) to have knowledge of the risk of rabies in humans compared to a value of less than Rp250,000 per dog. The final model had a good fit with the data (Hosmer-Lemeshow goodness-of-fit test p-value was 0.76) and no multicollinearity was found between the independent variables (highest Spearman’s rank correlation coefficient (ρ) was 0.36).

**Table 2:** Determinants of knowledge about the risk of rabies to human health (*rabies is a fatal disease in humans (yes/no*)) in the logistic multivariable regression model (n = 446).

Variables	OR (95% CI)	p-value
Regency:		
Manggarai	1.00	
Sikka	5.55 (2.33–13.18)	0.000
Having male dog:		
No	1.00	
Yes	0.41(0.18–0.96)	0.040
Economic value of dogs* (in Rupiah(Rp)^1^ per dog):		
≤ 250,000	1.00	
>250,000–500,000	2.74 (1.14–6.59)	0.024
> 500,000	0.60 (0.18–2.07)	0.419

OR = Odds ratio; CI = Confidence interval.

*The economic value of dogs was based on the owners’ estimation.

^1^The currency rate when the study was conducted, 1 February 2013: 1US$ = Rp 9,651.

The Hosmer-Lemeshow goodness-of-fit test p-value for this model was 0.76.

Knowledge of the prevention of rabies in humans was significantly associated with regency, economic value of dogs, and education level (Table 3). The odds of having knowledge of rabies prevention were higher among dog owners living in Sikka (OR = 3.44; 95% CI = 1.68–7.05), having a high educational level (OR = 4.64; 95% CI = 1.50–14.33), and having dogs with an average economic value between Rp250,000 and Rp500,000 per dog (OR = 2.94; 95% CI = 1.40–6.16) compared to a value of ≤Rp250,000 per dog. The Hosmer-Lemeshow goodness-of-fit test p-value for this model was 0.48, which indicates an adequate fit of the model to the data. There was no multicollinearity between the independent variables (the highest Spearman’s rank correlation coefficient (ρ) was 0.53).

**Table 3:** Determinants of knowledge about rabies prevention (*rabies in humans can be prevented (yes/no)*) in the logistic multivariable regression model (n = 446).

Variables	OR (95% CI)	p-value
Regency:		
Manggarai	1.00	
Sikka	3.44 (1.68–7.05)	0.001
Education:		
None	1.00	
Elementary school	2.17 (0.92–5.17)	0.079
Junior high school	3.37 (1.08–10.51)	0.036
Senior high school/University	4.64 (1.50–14.33)	0.008
Economic value of dogs* (in Rupiah(Rp)^1^ per dog):		
≤ 250,000	1.00	
>250,000–500,000	2.94 (1.40–6.16)	0.004
> 500,000	0.94 (0.30–2.96)	0.910

OR = Odds ratio; CI = Confidence interval.

*The economic value of dogs was based on the owners’ estimation.

^1^The currency rate when the study was conducted, 1 February 2013: 1US$ = Rp 9,651.

The Hosmer-Lemeshow goodness-of-fit test p-value for this model was 0.48.

The results of the logistic multivariable regression analysis (Table 4) on the level of knowledge of rabies control measures showed a significant association with the following factors: primary function of dogs, the level of dog owners’ income, and the geographical accessibility of the village. The odds of having a high level of knowledge of rabies control measures was higher in the following situations: for dog owners who lived in villages with good accessibility (OR = 2.14; 95% CI = 1.07–4.27) compared to poor accessibility, for dog owners who kept dogs as a source of income (economy) (OR = 3.18; 95% CI = 1.20–8.44) or as a guard of the house or property (OR = 3.44; 95% CI = 1.39–8.51) compared to the function of traditional ceremony, and for dog owners who had an income of more than Rp1,000,000 (OR = 3.02; 95% CI = 1.36–6.71) compared to an income of Rp500,000–1,000,000. The model fitted the data well (the Hosmer-Lemeshow goodness-of-fit test p-value was 0.80) and no multicollinearity was found between independent variables (the highest ρ was 0.37).

**Table 4:** Determinants of the level of knowledge of rabies control measures (*high > = 3 measures /low < 3 measures*) in the logistic multivariable regression model (n = 399).

Variables	OR (95% CI)	p-value
Primary function of dogs:		
Traditional ceremony	1.0	
Economy	3.18 (1.20–8.44)	0.020
Source of protein	3.60 (0.84–15.38)	0.084
Guard of house/property	3.44 (1.39–8.51)	0.007
Hunter/chaser of wild animals	1.65 (0.57–4.78)	0.353
Monthly income of dog owners* (in Rupiah(Rp)^1^):		
< 500,000	1.61 (0.98–2.66)	0.063
500,000–1,000,000	1.00	
> 1,000,000	3.02 (1.36–6.71)	0.007
Geographical accessibility of the village:		
Poor	1.00	
Average	1.36 (0.82–2.27)	0.227
Good	2.14 (1.07–4.27)	0.031

OR = Odds ratio; CI = Confidence interval.

*4 missing values.

^1^The currency rate when the study was conducted, 1 February 2013: 1US$ = Rp 9,651.

The Hosmer-Lemeshow goodness-of-fit test p-value for this model was 0.80.

### Uptake of control measures

**Control measures in dogs.** Respondents’ uptake of rabies control measures during the last fourteen years (1999–2012) are shown in Table 5. Fifty-six percent of respondents reported that at least one of their dogs had been vaccinated during the last fourteen years, the majority of which (92%) had had their dogs vaccinated during the 2012 campaign. Regarding culling as a control measure, 33% of the dog owners reported that at least one of their dogs had been culled during the last fourteen years. The majority of the culling was carried out in 1999 (45%) and 2010 (25%). Only 1% of the respondents reported that at least one of their dogs had been culled in 2012. Most often, these dogs were culled after having bitten someone or showing unusual behavior. In total, 12% of the dog owners had castrated at least one of their dogs. The main purpose given by the dog owners for castration was to keep the dogs close to home (prevent roaming away) in their function as guard of the house and property or as hunter to chase away wildlife. Castration of male dogs was carried out by the dog owners themselves or by their family.

**Table 5:** Uptake of rabies control measures to reduce human rabies cases in Sikka and Manggarai regencies during the period 1999–2012.

**I. Rabies control measures in dogs**	**Sikka (n = 300) n (%)**	**Manggarai (n = 150) n (%)**	**Total (N = 450) n (%)**
a.Dog vaccination	196 (65.3)	58 (38.7)	254 (56.4)
b. Culling of dogs	82 (27.3)	68 (45.3)	150 (33.3)
c. Castration of male dogs	34 (11.3)	19 (12.7)	53 (11.8)
**II. Rabies control measures in humans**	**Sikka (n = 67) n (%)**	**Manggarai (n = 22) n (%)**	Total (N = 89*) n (%)
a. Wound cleaning	(88.1)	16 (72.7)	75 (88.3)
b. Vaccine and/or immunoglobulin injection	35 (52.2)	15 (68.2)	50 (56.2)

*Number of dog owners that experienced a dog bite among one of their family members.

The uptake of vaccination and culling differed among regencies. The proportion of dog owners who had vaccinated their dogs was significantly (p<0.001) higher in Sikka (65%) than in Manggarai (39%) (Table 5). In contrast, the proportion of dog owners that had culled their dogs was significantly higher in Manggarai (45%) than in Sikka (27%) (p<0.001).

**Control measures in humans.** Of the 450 dog owners interviewed, 89 (20%) reported that at least one of their family members had been bitten by a suspected rabid dog during the last fourteen years. Of these 89 bite cases, 75 (84%) cleaned the wound, and 50 (56%) received vaccination. The level of uptake of these measures did not differ between regencies (p>0.05). Approximately 87% of the reported bite cases occurred during the period 2009–2012, of which 34% in 2012 alone.

**Uptake of the 2012 vaccination campaign.** During the 2012 vaccination campaign, 52% (234/450) of the dog owners had vaccinated at least one of their dogs (Table 1). This uptake proportion was significantly higher in Sikka (63%; 189/300) than in Manggarai (30%; 45/150) (p<0.001). The proportion of vaccination uptake was also significantly higher for owners of female dogs (59%; 87/147) than male dogs (49%; 147/303) (p<0.05). The vaccination uptake was significantly associated with the knowledge of the dog owners about rabies (p<0.001) and its control measures (p<0.05) (Table 6). The proportion of dog owners who had vaccinated dogs was higher for those who considered rabies a fatal disease (54%; 225/415) than for those who did not (26%; 9/35). Similarly, dog owners with a high level of knowledge of rabies control measures (59%; 161/272) tended to vaccinate their dogs compared to their counterparts (46%; 60/131). We found no significant association of vaccination uptake with the age and education level of the dog owner, presence of children in the household, or having male dogs (Table 1).

**Table 6:** Rabies knowledge of surveyed dog owners in Flores Island in relation to their uptake of the 2012 vaccination campaign.

Variables	Uptake 2012 vaccination campaign (N = 450)		
	**Yes**	**No**	
	**n**	**n**	**p-value**
Knew that rabies is a fatal disease in humans:			0.001
Yes	225	190	
No	9	26	
Knew that rabies in humans can be prevented:			0.000
Yes	221	182	
No	13	34	
Knowledge level of rabies control measures*:			0.010
High	161	111	
Low	60	71	

*The question was posed only to dog owners who knew that “rabies in humans can be prevented” (n = 403).

Differences were tested with a Chi square test.

**Multivariable model for the 2012 vaccination uptake.** Of the 13 independent variables that had an association (p-value less than 0.25) with the uptake of the 2012 vaccination campaign in the univariable analyses (Table 1 and Table 6), only five variables were retained in the final multivariable model. Regency, having female dogs for breeding, economic value of dogs, income of dog owners, and accessibility of the village were significantly associated with the uptake of vaccination (Table 7). Dog owners from Sikka were more likely to vaccinate their dogs (OR = 4.07; 95% CI = 2.30–7.20) than those from Manggarai. The dog owners who held female dogs for breeding had significantly higher odds to vaccinate their dogs (OR = 2.07; 95%CI = 1.31–3.27) compared with those who did not. Similarly, the dog owners who owned dogs with an economic value ranging between Rp250,000–500,000 tended to vaccinate their dogs (OR = 2.38; 95%CI = 1.36–4.17) compared with owners who valued their dogs at less than or equal to Rp250,000. Moreover, the uptake of vaccination was higher if the dog owners had a monthly income of more than 1 million Rupiah (OR = 2.39; 95%CI = 1.10–5.20) and lived in a village with a good accessibility (OR = 3.84; 95%CI = 1.92–7.67) compared with those having a yearly income less than Rp500,000 and who lived in a village with poor accessibility. The Hosmer-Lemeshow goodness-of-fit test p-value for this model was 0.85, which indicates the model fitted the data well. There was no multicollinearity between independent variables (the highest Spearman’s rank correlation coefficient (ρ) was 0.49).

**Table 7:** Determinants of the uptake of the 2012 vaccination campaign (*yes/no*) in the logistic multivariable regression model (n = 399).

Variables	OR (95% CI)	p-value
Regency:		
Manggarai	1.00	
Sikka	4.07 (2.30–7.20)	0.000
Having female dogs for breeding*:		
No	1.00	
Yes	2.07 (1.31–3.27)	0.002
Economic value of dogs** (in Rupiah(Rp)^1^ per dog):		
≤ 250,000	1.00	
>250,000–500,000	2.38 (1.36–4.17)	0.002
>500,000	0.24 (0.03–2.04)	0.191
Monthly income of dog owners*** (in Rupiah(Rp)^1^):		
< 500,000	1.00	
500,000–1,000,000	0.81 (0.47–1.39)	0.434
> 1,000,000	2.39 (1.10–5.20)	0.028
Geographical accessibility of the village:		
Poor	1.00	
Average	1.80 (1.09–2.97)	0.022
Good	3.84 (1.92–7.67)	0.000

OR = Odds ratio; CI = Confidence interval.

*Female dogs that had been giving birth during their life.

**The economic value of dogs was based on the owners’ estimation.

***4 missing values.

^1^The currency rate when the study was conducted, 1 February 2013: 1US$ = Rp 9,651.

The Hosmer-Lemeshow goodness-of-fit test p-value for this model was 0.85.

**Fig 2:**
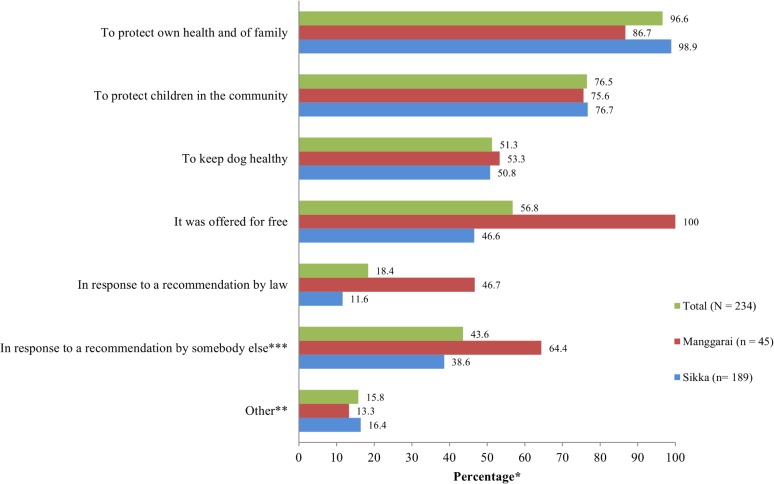
Reasons for joining the vaccination campaign of 2012. *Dog owners were allowed to provide more than one response; therefore, percentages of reasons do not sum to 100%; **To support the government’s campaign or in response to the fact that vaccinators were visiting at home; ***Neighbor, relative, family, and village leaders.

**Fig 3:**
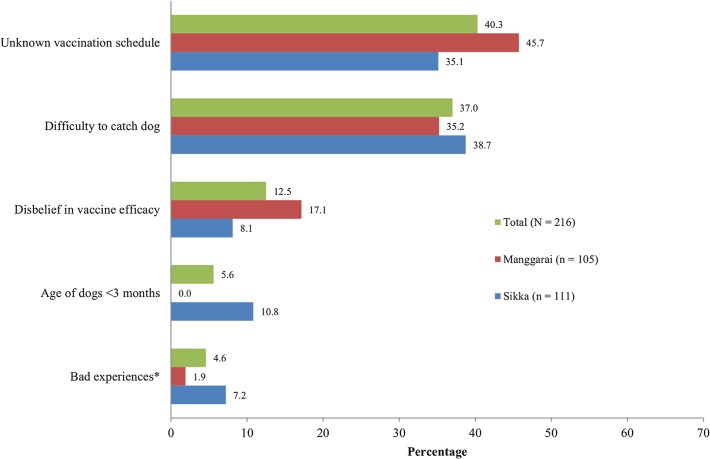
Main reason for not joining the vaccination campaign of 2012. *Bad experiences with earlier vaccinations reflected by the perception that it made the dogs less aggressive or less fertile or that it eventuated in the death of the dogs.

**Motivation to adopt the vaccination control campaign.** Reasons for having their dogs vaccinated against rabies in 2012 were given by the 234 dog owners who indicated that at least one of their dogs was vaccinated during this vaccination campaign. The most common reasons for the dog owners to vaccinate their dogs were to protect their own health and that of their family (97%) and to protect the children in their community (77%) (Fig. 2). For those dog owners who had not vaccinated their dogs (216 of 450 dog owners), the most important reasons for not joining were the lack of information about the schedule of the vaccination campaign (40%) and the difficulty to catch their dogs during the vaccination campaign (37%) (Fig. 3). Other reasons, of minor importance, included the lack of belief in the vaccine efficacy (13%), and the young age of the dog at the time of the vaccination campaign (6%).

## Discussion

The knowledge of dog owners in Flores Island about the risk of rabies to human health and about the possibilities to prevent the disease was generally high. This positive result might have been overestimated or biased due to the structure of the questions posed (“*do you know that rabies is a fatal disease*” and “*do you know that rabies in humans can be prevented*”). However, given the dog owners’ prompt responses on the subsequent open questions, e.g., *“which control measures are known to you in order to prevent rabies in humans”*, to which all dog owners provided a response, we expect that the structure of the questions did not influence the result substantially. This high knowledge level is comparable with findings of other studies conducted in South East Asian countries [22,32,33]. The high level of knowledge about the risk of rabies and its control might be due to the long history of rabies in these countries and the frequent coverage of human rabies cases by the mass media.

Vaccination of dogs against the rabies virus offers a safe and effective means to prevent rabies infection in humans [34]. Vaccination coverage should be at least 70% [1] to maintain the control of rabies between annual vaccination campaigns. Mass vaccination of dogs (70% of the estimated total number of dogs) in Bali Island, Indonesia, successfully decreased the human rabies incidence on that island by 74% [35]. In our study, around 52% of the dog owners had vaccinated at least one of their dogs in 2012. Real vaccination coverage (number of dogs vaccinated divided by the size of the total dog population) will be lower than the estimated 52%, as most households own multiple dogs, which makes it hard to handle them all at a single time during a vaccination campaign. The Sikka Regency estimated the vaccination coverage during the 2012 campaign to be around 58%. This recorded coverage rate, however, overestimated the real coverage as it did not account for the dogs and their owners that were not at home during the ‘house-to-house’ campaign. These non-registered dogs were estimated to represent approximately 30% of the total dog population [4]. These findings indicate that the real rate of vaccination coverage for the dog population in Flores Island is still far below the WHO recommended rate of 70% [1]. A targeted vaccination coverage of 70% is very important to maintain the overall herd immunity between campaigns above the threshold immunity coverage (e.g. 20–45%; [36]). This is especially relevant for Flores Island, where the dog population is characterized by a high turn-over rate (>45%) [37] and the vaccine used has a short duration of immunity. High quality, cell-culture vaccines are recommended for rabies control, such as Rabisin, which was used to effectively reduce the prevalence of dog and human rabies in Bali [35].

Relatively more dog owners in Sikka vaccinated their dogs than in Manggarai, whereas in Manggarai the proportion of dog owners that had culled dogs was higher than in Sikka. This difference reflects the different approaches used by the regencies to implement control measures. The animal health authority of Sikka has focused on vaccination of dogs as the main approach to control rabies in the regency, which is in line with the national campaign. Whereas the authority in Manggarai implemented culling of roaming dogs as an additional control measure alongside the national campaign. In 2010, for example, Manggarai conducted mass culling of 2,440 dogs (24% of the estimated total number of dogs in the regency), which were free roaming in the public area, regardless of the vaccination status of these dogs [38]. As a consequence, the size of the dog population in Manggarai reduced considerably [4]. The number of registered dogs during the vaccination campaign of 2012 was six times lower than in Sikka, even though the size of the human population in both regencies was comparable. A positive impact of culling is the removal of all potentially exposed dogs in infected villages, thereby reducing the transmission of rabies between dogs and decreasing the risk of rabies for humans [10]. The culling of free roaming dogs was, however, less acceptable for the local community in comparison to the vaccination campaign [4]. This resulted in unintended negative consequences [39] in which the dog owners hid or moved their dogs to another village during the incubation phase of rabies [9,40]. In this context, the OIE and other international animal health related organizations (e.g. WHO, WSAVA, and GARC) do not recommend culling as a rabies control measure [27,41,42,43]. Culling (i.e. the killing of dogs regardless of their health status) is not effective in controlling rabies [44] and can be counterproductive [43], as previously vaccinated dogs may also be culled.

The difference in vaccination uptake between the regencies might also be due to the intensity of local community support for the control campaign [45]. Religious and village leaders in Sikka participated actively in encouraging dog owners to vaccinate their dogs, whereas this was not the case in Manggarai. The encouragement of community leaders may have stimulated dog owners to increase their efforts to catch and restrain the dog to be vaccinated. In addition, the vaccination campaign in Sikka coincided with the national celebration of World Rabies Day on 8^th^ October 2012 in Maumere. During this celebration, religious and village leaders were invited to join the event to share experiences on rabies control measures in their villages. Our findings suggest that the involvement of local communities in rabies control activities can be important to implement rabies control measures successfully in a regency. Moreover, a good collaboration among sectors, such as public health and veterinary authorities, is also important, as was reported from Latin America [1].

Theoretical and empirical evidence suggests that reducing population density through sterilization, which includes castration of male dogs, does not reduce disease transmission [36,44]. There is limited, equivocal empirical evidence that in open, dynamic populations mass sterilization extends vaccination by reducing the number of new susceptible dogs entering the population through reducing local births [46,47,48]. Nonetheless, castration of males has been encouraged by the local authority since 2000, as a method to limit the number of free roaming dogs within villages in Flores and restrict male dog behavior (such as dispersal and fighting) that facilitates the spread of rabies [1]. Humane dog population management, which includes sterilization and the provision of basic dog health care, is currently recommended as a supplementary measure to mass vaccination programs [41], and for this reason we have estimated the prevalence of castration. Only 12% of dog owners reported that at least one of their male dogs had been castrated during the period 1999–2000. This low uptake might be due to the lack of skill to castrate dogs as castration of male dogs was carried out by the dog owners themselves or by their family. Another reason could be attributed to the dog owners’ preference, especially those who keep female dogs for breeding, to have a male dog without castration.

Dog owners, both in Sikka and Manggarai, had a high level of knowledge about the preventive measures to be taken after being bitten by a suspected rabid dog. However, a high level of knowledge of rabies and its prevention does not guarantee a high uptake of proper treatment after exposure. In our study, less than 60% of patients (89 reported bite cases in humans) went to the medical center to seek proper medical treatment, even though the majority of the community knew that rabies in humans is fatal and can be prevented by a series of vaccine injections after exposure. Most of the people in Flores Island that died after being bitten by a rabid dog did not receive any post exposure treatment (Purnama, personal communication, 2014). In addition to the level of knowledge, socio-economic factors such as income level and distance to the nearest rabies-treatment center can contribute significantly to the decision to adopt the appropriate treatment [49]. People living in rural areas, far from any rabies-treatment center, may not have access to prompt and appropriate treatment [49]. Even if the treatment is provided for free (e.g. costs of human rabies vaccines and physician costs are paid by the individual Regency Governments of Flores Island), costs associated with travel to and from the rabies-treatment center and income loss during the treatment [50] could prevent dog-bite victims from seeking medical care.

The distribution of reported control measures in dogs and bite cases in humans over the previous fourteen years may have been influenced by recall bias, reflecting the extensive time frame posed in the research questions. However, the results give an overview of the uptake of rabies control measures over the previous fourteen years and could be used for better planning of rabies control in the future.

In the univariable analysis, knowledge of rabies and its control measures was significantly associated with the 2012 vaccination uptake by dog owners. However, in the multivariable analysis this association was no longer significant. This indicates that the level of rabies knowledge did not have a direct effect on the uptake of the 2012 vaccination campaign. An important factor associated with vaccination uptake was the accessibility of the village. Uptake of the vaccination campaign was four times more likely for dog owners living in a village with good infrastructure than for those living in more remote villages with a poor road infrastructure. This is an interesting finding, as this factor has not been studied before. The accessibility of a village might be related to the transfer of knowledge from animal health authorities (especially the distribution of vaccination schedule information). Informal discussions during the survey with dog owners in the less accessible villages revealed that many of these dog owners became aware of the vaccination campaign only on the day it was conducted. This is in line with our survey results, which showed that one of the main reasons for not joining the vaccination campaign was the lack of information about the vaccination campaign. As a consequence, dog owners were not at home when the vaccinators arrived. This corresponds with the study results of Durr et al [16], in which 26% of the surveyed dog owners did not join the vaccination campaign as they were not aware of the time schedule. In Flores Island, it is common practice for the notification letter about the vaccination schedule to be sent to the village leader through public transportation. Given the poor accessibility of the more remote villages due to natural barriers, (especially in the rainy season, September-April) these notifications do not always reach the villages on time. As a result, dog owners in less accessible villages are less informed about the vaccination campaign. This finding suggests that targeted distribution of vaccination campaign information within these villages is an effective and practical way to increase the uptake of rabies vaccination in the future. Effective channels for the distribution of information about the vaccination schedule, prior to the visit of the vaccination team, could be through elementary school teachers [51], and church and village leaders.

The second important reason given by dog owners for not joining the vaccination campaign was the inability to handle and restrain their dogs (37%). This reason was given more frequently in our study than reported in studies from other endemic rabies countries [18,20,52]. The difference could be due a different relationship between humans and dogs in those countries. Dogs in Flores are never restricted and roam freely within the village, so the interaction between owners and their dogs is very low. Dogs in Flores Island have a primary function as guard dogs. This type of dog is more aggressive and difficult to handle compared with companion dogs. This suggests that educating the vaccinators and dog owners about dog behavior and the safe handling of dogs might improve vaccination coverage whilst limiting the risk of being bitten. Alternatively, training teams of government dog catchers, similar to Bali [35], may be required to increase vaccination coverage if owners are unable to catch and restrain their dogs.

Our study highlighted the association between keeping female dogs for breeding and the uptake of vaccination. The dog owners who had female dogs for breeding purposes were more likely to join the vaccination campaign. The perceived value of the dog may have increased the dog owners’ effort to catch and restrain the dog to be vaccinated. Other reasons that might have contributed to this association are related to the relatively longer life span and lower turn-over rate of female dogs. In the event of a traditional ceremony in which dog meat is needed, dog owners in Flores prefer to cull male dogs and keep female dogs for breeding. A reproductive female can produce puppies until the age of 11 years (reproductive lifespan) [53]. Therefore, dog owners get more benefit from vaccinating reproductive female dogs.

Furthermore, our study found that dog owners who valued their dogs at less than Rp250,000 were less likely to join the vaccination campaign compared to dog owners with dogs valued between Rp250,000 and Rp500,000. This result might be due to the age of the dogs. The majority of the dogs valued at less than Rp250,000 were younger than one year old. It is well documented that dogs younger than one year are less likely to be vaccinated by their dog owners [14,18,54,55], putting this cohort at a higher risk for contracting rabies [56]. This is the result of a common perception that this cohort is too young to be vaccinated [20,55]. Vaccine manufacturers indicate that rabies vaccine can be effectively administered to dogs at as early as 3 months of age. Therefore, the vaccination campaign in the future should place additional emphasis on this unvaccinated cohort of dogs, aged between 3 and 12 months. In addition, community education efforts should be focused on dog owners who have a female dog for breeding, encouraging them to vaccinate their young dogs before selling them. Dog owners with a high income (more than 1 million Rupiah per year) had a higher probability of having their dogs vaccinated than dog owners with lower income levels. This finding is similar to the result of a study by Beran [40]. It is common practice in Flores Island for people with a good income to hire other adults (particularly from their family) to take care of their children and home. As a consequence, there will always be an adult person present at the home to handle the dogs during vaccination. This may explain our finding that dog owners with a higher income were more likely to vaccinate their dogs.

### Conclusions

The level of knowledge of dog owners in Flores Island about rabies and its control measures was high, but not associated with the uptake of the vaccination campaign of 2012. Overall, the uptake rate of the 2012 vaccination campaign was relatively low (52%) and differed between regencies. Geographical accessibility is one of the important predictors of vaccination uptake among dog owners. Targeted interventions in those villages with poor accessibility may increase the vaccination uptake in the future. These interventions should include: (1) an effective system for distributing information so that dog owners are provided with timely information on the vaccination schedule, for instance through elementary school teachers, and church and village leaders; and (2) the provision of dog owners and vaccinators with a technique or skill to catch and restrain dogs.

## Supporting Information

S1 Checklist STROBE Checklist. (DOC)
